# Spillover effects of food recalls: A milk recall scenario experiment in China

**DOI:** 10.1038/s41538-022-00139-1

**Published:** 2022-04-22

**Authors:** Na Hao, Yi Zhang, Qiujie Zheng, Michael Wetzstein

**Affiliations:** 1 School of Economics, Beijing Technology and Business University, Beijing, 100084 China; 2 School of Economics, Guizhou University, Guiyang, 550025 China; 3 Maine Business School, University of Maine, Orono, ME 04469 USA; 4 Department of Agricultural Economics, Purdue University, West Lafayette, IN 47907 USA; 5 Department of Agricultural and Applied Economics, University of Georgia, Athens, GA 30606 USA

**Keywords:** Economics, Economics

## Abstract

Food recall is a major ingredient in food safety with existing literature focusing mainly on its direct impacts. Few studies focus on possible spillover effects. It is hypothesized that food recalls have a spillover effect on the recalled brand and purchase channel. As a test of this hypothesis, a 2-purchase channel by 3-recall strategy scenario experiment was conducted on spillover effects of a milk recall in Beijing, China. The results indicate that food-safety scares have significant negative impacts on consumers’ purchase intention on the recalled brand and purchase channel, and the impacts are more significant for online than offline marketing. However, voluntary recalls by online firms help mitigate these negative effects and restore consumers’ purchase intention more than offline voluntary recalls. An online food incident creates an issue of trust toward general online platforms. Online vendors should take greater care in guaranteeing food safety and actively take restorative actions such as voluntary recalls after a food safety incident. Results provide empirical evidence for industry organizations and governments to stipulate a strict food safety and incident resolution system for e-commerce.

## Introduction

Food safety has always been a public concern across countries. At the start of 2021 alone, American company El Abuelito Cheese recalled multiple cheese products^1^; International Golden Foods, Inc (IGF) recalled specific lot codes of the Al kanater brand tahini because of possible health risk^2^; Three Squirrels, a brand popular with youth in China, recalled its pine nuts due to excess hydrogen dioxide^3^. Food safety incidents pose consumer health risks and trust issues, and can cause undue panic. Governments and firms often recall contaminated foods to mitigate such negative impacts. Since the food supply chain comprises multiple stages and components that are interconnected, the impact of a food recall crisis may go beyond the recalled product. Once a food recall crisis occurs, consumers may not only be reluctant to continue purchasing the recalled product, but are also not willing to purchase other products of the recalled brand or using the purchasing channel for some time, i.e., spillover effects. In addition, with online food shopping gaining popularity over the recent years, food product information distribution and consumers’ shopping preferences online are different than those in the traditional brick-and-mortar stores. Consumers’ responses to a food recall crisis through online purchasing channel may be different than offline, a factor needs to be taken into consideration in measuring the food recall impact.

The goal of this paper is to measure the spillover effects of food recalls by investigating consumers’ attitudes toward the recalled food product, other products of the same brand, and purchase channel after a recall crisis, using milk as an example. Milk, an essential nutritious food, has been recalled frequently for safety issues. Milk has many alternative brands for consumers to choose and hence is a good candidate to study for the food recall effect. Consumers’ attitudes are the essential factor to measure the effectiveness of a food recall^4^. We focus on three main issues in a food recall incident. First, we study consumers’ product brand choice after a recall crisis to explore the impact of the recall on sales of the affected product. Second, we study consumers’ willingness to purchase other products of the same brand to explore whether the brand reputation across all its products is affected by the recall. Finally, we study how soon the consumers will use the involved purchasing channel again to purchase the product to explore whether the recall impacts the purchase channel, especially the online platforms, such as JD.com and Alibaba’s Taobao.

In addition, food recalls happen in different situations. For example, a food recall may be initiated by different agencies (such as news media, food companies, and government) and through alternative purchasing channels (such as online platforms and stores). Different recall types may have different effects when food recalls occur^5–7^. Especially, the rapid development of e-commerce is changing the food consumption pattern, as more consumers tend to purchase food through e-commerce platforms^8,9^. Taking into account the purchase channels and recall types, we designed a two-purchase channel (*Online* vs. *Offline*) by three-recall strategy (*News media disclosure*, *Voluntary firm recall* vs. *Mandatory government recall*) scenario experiment to investigate the impact of different recall types and purchasing channels on consumers’ purchase intention in the three aspects mentioned above. Next, we review food recall issue and formulate hypotheses based on the literature.

Food recalls aim to withdraw unsafe food from the market, solving a serious problem in food safety^10^. They can be categorized into two types based on agencies who request the recall: voluntary recall by firms and mandatory recall by government^11^. According to the Food Safety Law and Provisions on Administration of Food Recall in China, voluntary recall occurs when food producers detect a problem, accept fault, voluntarily stop producing and selling the affected product, and inform government authorities and the public^12,13^. Voluntary recalls may fail due to consumer mistrust, government bureaucracy, and recall costs incurred by firms such as brand damage^12^. When firms fail to recall products or intentionally conceal the existence of an incident, government authorities may mandate a recall^10^. In practice, mandatory recalls dominate, with only a few Chinese firms opting to voluntarily recall^11^.

Food recalls are primarily driven by government^14,15^, however, news media has an important and complementary role in food safety governance^16^ and many food incidents were revealed by the media^17,18^. Governments and firms often in a hegemonic position may collude to suppress a food safety incident^19^. The news media are more flexible in disclosing food safety incidents^5,17^ and can trigger and amplify public concerns over food safety risks^20,21^. As a result, news media and the public urge governments and firms to solve the safety problem or recall the affected products^19^. One case of news exposure is when Shanghai’s *Oriental Morning News* broke the “melamine scandal” of Sanlu milk after consumers in vain complained to the government and the Sanlu Group. Media reported melamine-tained milk powder led to six infant deaths and over 300,000 sickened^22^. The incident has cast a long-lasting shadow on Chinese dairy industry causing a plummeting in consumers’ purchase intentions^23^. Thus, we include voluntary recall by the firm, mandatory recall by government, and news media exposure in our scenario design.

The literature has investigated the impacts of food recalls on the affected firms from the perspective of stock prices^24–26^. Other efforts have focused on consumer reactions to food recalls^6,27^. Consumers may overreact to food safety incidents, which may result in shunning unaffected products of the same firm or similar products from other firms^27–30^, i.e., spillover effects. For a comprehensive assessment, it is important to examine the impact of recalls in a broader scope rather than simply focus on the affected product. Research has analyzed the spillover effects caused by recalls and findings vary. Some indicate that recalling exerts negative spillover effects within the same brand family^31^ but has no significant effect on competing brands^32^. In contrast, some argue that the negative effects occur both in the recalled brand and its rivals, and that the effects are the strongest among brands within the same country^33^.

Based on this literature, we hypothesize:

*H1*. Food recalls not only affect the recalled milk but may also produce a negative spillover effect on other products of the same brand.

One objective of a food recall is to recover consumers’ positive purchase intention^7^. This may be more challenging for online firms if consumers have less trust in online commodites due to uncertainty^34,35^. It is generally more challenging to retain loyal consumers in the online than offline market^36,37^. Recalls of food sold online may have a different level of spillover effect magnitude than recalls of food sold offline.

We then hypothesize:

*H2*. The magnitude of food recall spillover effects varies by the purchase channel.

Research also has investigated the impact of different recall types on consumers’ purchasing decisions^6,38^. Passive strategies taken by firms, government mandated recalls, or news media exposure, have negative impacts on corporate image and consumer loyalty and purchase intention^20,38^. Voluntary recalls initiated by a firm may help consumers and companies to recover consumers’ purchase intention^6,7,38–40^. This recovery may depend on the proactive nature of the recall and associated compensation^6^. The seriousness and openness of a recall might affect if and how soon consumers will purchase the recalled product again^41^.

Based on this literatue, we hypothesize:

*H3*. Voluntary recall can alleviate the negative effects of food recalls on the recalled product, the other products of the same brand, and the purchase channel.

## Results

### Summary statistics

The survey was conducted in January 2020 and collected 360 responses with summary statistics listed in Table 1. For milk brand choice, the percentages of those who choose not to buy milk now, buy other brand milk or the recalled brand milk are 8.06%, 83.61% and 8.33%, respectively. The overall attitude of most consumers is to reject the recalled milk, confirming the negative impact of the recall. In terms of other products of the recalled brand, 33.33% of participants are willing to buy them in the near future and the remaining 66.66% are not. This suggests most consumers are cautious of the other products of the recalled brand. As for the duration of purchasing milk from the original channel, ranging from more than three months to one week, the percentages are 43.61%, 12.78%, 15.23%, and 28.33%, respectively. It indicates food recall has an indirect influence on the original purchasing channel.

**Table 1:** Survey questions and summary statistics.

Variables Name	Description	Statistics
*Dependent Variables*		
Milk_brand	$$\left\{ {\begin{array}{*{20}{l}} {0,may\,not\,buy\,milk\,for\,the\,time\,being} \\ {1,milk\,of\,other\,brands} \\ {2,the\,recalled\,brand} \end{array}} \right.$$	0: 8.06% 1: 83.61% 2: 8.33%
Other_products	$$\left\{ {\begin{array}{*{20}{l}} {0,if\,no} \\ {1,if\,yes} \end{array}} \right.$$	0: 66.67% 1: 33.33%
Channel	$$\left\{ {\begin{array}{*{20}{l}} {1,Not\,willing\,to\,buy\,over\,the\,following\,three\,months} \\ {2,Willing\,to\,buy\,one\,to\,three\,months\,afterwards} \\ {3,Willing\,to\,buy\,in\,the\,next\,month} \\ {4,Willing\,to\,buy\,in\,the\,next\,week} \end{array}} \right.$$	1: 43.61% 2: 12.78% 3: 15.23% 4: 28.33%
*Main Variables*		
Online	= 1 if purchase milk online; = 0 otherwise	0: 50% 1: 50%
Offline	= 1 if purchase milk offline; = 0 otherwise	0: 50% 1: 50%
News	= 1 if expose the milk with safety issues by news media; = 0 otherwise	0: 67% 1: 33%
Voluntary	= 1 if recall the milk with safety issues by the company; = 0 otherwise	0: 67% 1: 33%
Mandatory	= 1 if recall the milk with safety issues by the government; = 0 otherwise	0: 67% 1: 33%
*Other Variables*		
FoodonlinefreqH	= 1 if buy food online frequently (once a week at least); = 0 otherwise	0: 41% 1: 59%
MilkfreqH	= 1 if buy milk frequently (once a week at least); = 0 otherwise	0: 32% 1: 68%
WorryH	= 1 if highly worried; = 0 otherwise	0: 4% 1: 96%
Price	Levels 1-5 indicating importance, “1” for very unimportant, “5” for very important.	Mean 3.64 Std.Dev. 0.88
Brand	Levels 1-5 indicating importance, “1” for very unimportant, “5” for very important.	Mean 3.94 Std.Dev. 0.91
Age	The age of the respondents.	Mean 33.98 Std.Dev. 10.38
Male	=1 if male; = 0 if female	0: 52% 1: 48%
Edu	Years of schooling.	Mean 15.89 Std.Dev. 1.57
Income	Household annual income (10 thousand RMB)	Mean 20.06 Std.Dev. 12.86
Have_old	=1 if seniors; = 0 otherwise	0: 76% 1: 24%

Source: author’s survey, 2020.

*Voluntary* and *Mandatory* are dummy variables denoting recalling the unsafe milk by the company and the government, respectively, with exposure by news media (*News*) as the base. *Online* and *Offline* denote purchasing milk from online or offline channels, with *Offline* as the base. As indicated from Table 1, participants are distributed evenly across the two channels and three recall types.

The majority of respondents generally make online purchases frequently, with 59% reported purchasing food online at least once a week. Moreover, 68% of participants said they purchase milk at least once a week. Price and brand are the primary factors that participants value when purchasing milk. A comparison between the means of *Price* (3.64) and *Brand* (3.94) indicates that participants may pay more attention to milk quality than price. In addition, 96% of the participants said they would be worried about their health if they had consumed recalled milk. This indicates participants have a high level of safety awareness in milk consumption.

Demographic variables include gender, education, age, income, and elderly household members are also listed in Table 1. We collected 360 observations, covering the participants 18 years or older with an average age of 33.98. Males account for 48.06% and females 51.94%. Participants on average have 15.89 years of schooling with education levels range from junior/vocational college to master degree. The average family annual income is approximately 200 thousand yuan and 24% of households have at least one elderly member.

### Probit regression results

Two models analyze the spillover effects of food recall. The first model explores the effects of different influencing factors on participants’ response to the recall brand and the original purchasing channel after the recall. The second considers the interaction terms between *Online* and recall types to investigate whether online recalls have different effects.

### Spillover brand effects

In order to study the spillover effects of the food recall on the recalled brand, we first investigate participants’ choice of milk after a recall crisis. We employ a multinomial probit regression by using continuing to purchase recalled milk as a base. Results of choosing to not purchase any milk and to purchase another brand’s milk are listed in Table 2. *Online* in models 1 and 2 are significantly positive for the choice of not purchasing any milk at the 5% and 1% level, respectively. This indicates if participants purchased milk on an online platform and the milk is subsequently recalled, they are more likely to choose not to purchase milk in the near future. This is consistent with a previous study which found that the recall incident online not only affects the sales of the recalled milk, but also has a negative spillover effect on other milk brands^33^. Internet-based trust is fragile, and once a food safety incident occurs, online consumers’ purchase intention may be harder to restore.

**Table 2:** Multinomial probit model results of milk choice.

	Milk_brand			
	Model (1)		Model (2)	
	No purchase	Other brands	No purchase	Other Brands
Online	0.717**	0.374	1.273*	0.778
	(0.364)	(0.284)	(0.727)	(0.595)
Voluntary	−1.002**	−0.488	−1.016	−0.326
	(0.494)	(0.354)	(0.775)	(0.460)
Mandatory	0.245	−0.267	0.713	−0.026
	(0.440)	(0.366)	(0.620)	(0.474)
Online*Voluntary			−0.448	−0.448
			(0.737)	(0.737)
Online*Mandatory			−0.638	−0.632
			(0.769)	(0.769)
FoodonlinefreqH	−1.138***	−0.811**	−1.154***	−0.813**
	(0.425)	(0.347)	(0.427)	(0.347)
MilkfreqH	−0.309	0.040	−0.302	0.038
	(0.421)	(0.337)	(0.425)	(0.340)
WorryH	0.898	0.625	1.007	0.656
	(0.820)	(0.589)	(0.836)	(0.594)
Price	−0.296	−0.224	−0.329	−0.233
	(0.210)	(0.163)	(0.213)	(0.164)
Brand	0.053	0.204	0.028	0.197
	(0.202)	(0.162)	(0.206)	(0.164)
Age	−0.011	0.004	−0.011	0.004
	(0.019)	(0.015)	(0.019)	(0.015)
Male	−0.074	0.177	−0.089	0.174
	(0.373)	(0.291)	(0.377)	(0.294)
Edu	−0.009	0.080	−0.004	0.084
	(0.128)	(0.101)	(0.129)	(0.101)
Income	−0.013	0.002	−0.015	0.001
	(0.016)	(0.012)	(0.016)	(0.012)
Have_old	−0.241	−0.156	−0.206	−0.132
	(0.424)	(0.311)	(0.427)	(0.315)
_cons	1.584	0.409	1.393	0.268
	(2.294)	(1.802)	(2.337)	(1.833)
Observations	360		360	
Wald chi^2^	34.32		34.22	

Note: standard errors are reported in (). ***, ** and * denote the coefficient estimates are statistically significant at 1%, 5% and 10% levels, respectively.

Initiator also matters. In model 1, *Voluntary* is significantly negative for the choice of not purchasing any milk at the 5% level, indicating that participants are willing to repurchase recalled milk when the company voluntarily recalls. This is consistent with the recall literature showing proactive strategies by firms can help restore brand image and consumer trust behavior^38^. However, after adding interaction terms in model 2, the effect of *Voluntary* on the recalled milk is not significant. Further, Online*Voluntary is negative but statistically insignificant for the choice of not purchasing any milk. The results reinforce the fact that online trust is harder to restore. This suggests firms should put effort into maintaining their image, especially for online customers. *Mandatory* in the two models are insignificant for the choice of not purchasing any milk. This indicates the public trust in government competence to solve food safety issues has no positive impact on the recalled milk. This result confirms that if firms take passive strategies and wait until the government initiates the recall, it will hurt brand image and consumer loyalty^20,38^ and results in poor reputation and lost sales.

In addition, *FoodonlinefreqH* is significantly negative for the choice of not purchasing any milk and the choice of purchasing another brand’s milk at the 1% and 5% levels, respectively. This indicates consumers who are used to buying food online frequently may have higher loyalty and are more willing to continue to purchase the recalled milk. This is also consistent with the existing literatures that high user consistency may lead to higher loyalty^41,42^.

As hypothesized, recalling unsafe milk not only affects the recalled milk but may also affect other products of the same brand. Testing this hypothesis, we analyze the spillover effects of a recall on other products of the brand (Table 3). Given other products are not the direct subject of the recall and may suffer lagged effects, we investigate consumers’ willingness to purchase other products of the recalled brand in the coming month. Some consumer characteristics have a different impact on other products of the same brand. Similar to the results reported in Table 2, *FoodonlinefreqH* also reflects a significantly positive effect on other products of the same brand at the 5% level. But *WorryH* is significantly negative at the 1% level in both models, suggesting that the more worried consumers are about their health, the less likely they are to buy other products of the same brand. Supporting the hypothesis *H1*, results indicate recall produces a negative spillover effect on the other products of the same brand. Therefore, when faced with a recall, the priority of the firm is to ease consumer fears and restore consumers’ purchase intention. This confirms previous literature that spillover effects occur in the same brand^31^.

**Table 3:** Probit model results of whether purchasing other products of the same brand.

	Other_products	
	Model (1)	Model (2)
Online	0.047	−0.371
	(0.141)	(0.252)
Voluntary	0.400**	0.092
	(0.174)	(0.244)
Mandatory	0.109	−0.185
	(0.176)	(0.248)
Online*Volulntary		0.629*
		(0.347)
Online*Mandatory		0.594*
		(0.355)
FoodonlinefreqH	0.324**	0.340**
	(0.160)	(0.161)
MilkfreqH	0.086	0.091
	(0.168)	(0.169)
WorryH	−1.054***	−1.088***
	(0.374)	(0.384)
Price	0.112	0.116
	(0.081)	(0.082)
Brand	0.006	0.009
	(0.083)	(0.083)
Age	0.006	0.007
	(0.007)	(0.007)
Male	−0.196	−0.200
	(0.144)	(0.146)
Edu	0.040	0.039
	(0.050)	(0.050)
Income	0.003	0.003
	(0.006)	(0.006)
Have_old	−0.242	−0.271
	(0.168)	(0.170)
_cons	−1.078	−0.874
	(0.941)	(0.954)
Observations	360	360
Pseudo R^2^	0.057	0.066
LR chi^2^	26.33**	30.41**

Note: standard errors are reported in (). ***, **, and * denote the coefficient estimates are statistically significant at 1%, 5% and 10% levels, respectively.

In fact, *Voluntary* is significantly positive for *Other_products* at the 5% level in model 1, indicating if a firm voluntarily recalled milk, consumers are more likely to purchase other products from the brand. Recalling voluntarily helps companies restore their image. Voluntary recall is the embodiment of a company’s commitment to taking responsibility, which results in consumers’ willingness to hold a tolerant attitude toward its other products.

After adding the interaction terms in model 2, Online*Voluntary and Online*Mandatory are significantly positive at the 10% level. This suggests that compared with news media exposure, recalls initiated by a firm or the government can exert higher positive effects on purchasing other products of the brand through online channel. A responsible firm image (voluntary recall) or government credibility (mandatory recall) are more effective online than offline for other products of the recalled brand. Therefore, online vendors especially should recall unsafe products voluntarily to protect their reputation.

### Spillover purchasing channel effects

Due to their virtual and intangible characteristics, it is more difficult for online platforms than brick-and-mortar stores to gain consumers’ trust. Given that it may take time for online consumers to restore their confidence in the platform, we collected information on how soon consumers will resume milk purchases on a platform (i.e., online or in-stores) after a recall. *Channel* is an ordinal variable with a time span from one week to more than three months, and an ordered probit regression is performed.

The results are listed in Table 4. *Online* in model 1 is significantly negative at the 5% level, indicating that it will take longer to restore consumers’ purchasing confidence in online platforms than offline stores. But, *Voluntary* is significantly positive at the 1% level, suggesting that voluntary recalls by firms help shorten the time for consumers to restore confidence in purchasing milk from the original purchasing channel. *Mandatory* is significantly positive at the 10% level. Comparing the coefficients and significance of *Voluntary* and *Mandatory* indicates that voluntary recalls generate greater benefits. These results support the hypothesis *H2*.

**Table 4:** Ordered probit model results of milk purchasing platform.

	Channel	
	Model (1)	Model (2)
Online	−0.296**	−0.614***
	(0.122)	(0.217)
Voluntary	0.497***	0.235
	(0.151)	(0.208)
Mandatory	0.251*	0.082
	(0.151)	(0.206)
Online*Voluntary		0.557*
		(0.302)
Online*Mandatory		0.374
		(0.304)
FoodonlinefreqH	0.155	0.166
	(0.136)	(0.137)
MilkfreqH	0.206	0.213
	(0.145)	(0.145)
WorryH	−0.774**	−0.769**
	(0.312)	(0.313)
Price	0.091	0.092
	(0.070)	(0.071)
Brand	0.000	0.002
	(0.070)	(0.070)
Age	−0.001	−0.001
	(0.006)	(0.006)
Male	−0.172	−0.181
	(0.125)	(0.125)
Edu	0.010	0.006
	(0.043)	(0.043)
Income	0.005	0.005
	(0.005)	(0.005)
Have_old	0.179	0.148
	(0.143)	(0.145)
/cut1	−0.047	−0.228
	(0.811)	(0.818)
/cut2	0.294	0.117
	(0.811)	(0.818)
/cut3	0.734	0.557
	(0.812)	(0.819)
Observations	360	360
Pseudo R^2^	0.037	0.041
LR chi^2^	33.78***	37.32***

Note: standard errors are reported in (). ***, **, and * denote the coefficient estimates are statistically significant at 1%, 5%, and 10% levels, respectively.

For the model 2 regression, *Online* is significantly negative at the 1% level. However, Online*Voluntary is significantly positive at the 10% level. This indicates that voluntary recalls by firms play a pivotal role in restoring consumers’ purchase intention in online e-commerce platforms. Coupled with the results in model 1, *Voluntary* recalls have greater positive impacts on online platforms than offline brick-and-mortar stores. The responsible image created by voluntary recalls is magnified on online platforms, thus more effectively restoring consumers purchase intention. In fact, consumers’ purchase intention is a matter of trust in the products and the channels. According to trust transfer theory, trust can transfer from a trusted entity to another one^43^. In our case, consumers’ trust in firms can transfer to the purchase channel, especially online platforms. Specifically, according to the results, online platforms would suffer more severe adverse impact when food recalls occur, compared to offline platforms. But if the firms recall voluntarily, consumers’ trust on firms would transfer to involved online platforms, mitigating the negative impact on online platforms. The recall’s negative impact on online platforms vs. offline ones differentiates due to the firms’ voluntary recall action.

The positive results of *Voluntary* in the three tables support hypothesis *H3* that voluntary recalls play an essential role in restoring consumers’ purchase intention to the recalled milk, the other products of the same brand and the purchase channels. A voluntary recall is a win–win for companies and the channels, especially for online companies and online platforms. Online platforms should also monitor e-merchants to ensure food safety and respond proactively in the event of a food safety incident.

## Discussion

Frequent food safety incidents may not only cause consumers to lose their trust in the affected food product but also other products of the brand, and even the whole industry. Using milk as an example, we analyze the impacts of food recalls and reveal the following findings.

A safety incident exerts a negative effect on the affected brand. After a recall incident, consumers tend to avoid buying the recalled brand products. However, consumers are more likely to repurchase the recalled brand products if firms choose to recall unsafe products voluntarily, especially for online consumers. This supports the positive effect of voluntary recalls. Firms, especially in e-commerce, should consider taking responsibility for food safety.

Furthermore, e-commerce platforms may be more susceptible than offline brick-and-mortar stores from a food recall. It is more difficult to restore consumer confidence in purchases from online platforms than from offline stores. Voluntary recalls are more effective on e-commerce platforms than on offline stores, significantly mitigating negative impacts on the recalled brand and platform.

With regard to the survey and data, some limitations exist. We only used milk as the object of studying the spillover effects of food recalls, which may not generalize to other foods. Other food products that have experienced recalls might be considered in future research to confirm the robustness of results. Given the data were collected in Beijing only, where consumers may be more concerned about food safety, limitation exists in generalizing the results to other regions.

## Methodology

### Survey Design

To evaluate the spillover effects of food recalls, we conducted a scenario experiment in 2020 on Beijing consumers’ response in different recall scenarios. We commissioned a credible online marketing research company, Dynata, to distribute questionnaires and collect data. As no access to receive ethical approval and no such requirement in China at the time, we didn’t receive approval from relevant ethical regulations. We included a brief consent form at the beginning of survey describing that the survey was for research purpose and anonymous, collected information would be kept confidential, and participation was voluntary. If the respondent agreed to participate, he or she would click the start button. The consumer survey included only routine questions and there were no physical or psychological interruptions to the subjects and no risks to the participants.

In the survey, we designed recall scenarios by varying the initiators of the recall (i.e., government, firm, or news media) and the channels from which consumers purchase milk (i.e., online e-commerce or offline brick-and-mortar stores). We designed six scenario treatments (2-purchase channel × 3-recall strategy) and randomly and equally assigned a total of 360 participants to each of the treatments. Based on previous literature, in the survey we included questions about how often participants purchased food online^35,44^; how often they purchased milk^45,46^; whether participants would be worried about their health if they had consumed a recalled food^6,40,47^; how important is price to participants’ milk purchasing decision^48,49^; how important is brand to participants’ milk purchasing decision^48,50,51^. After a range of questions on purchasing habits, risk perception, product characteristics and demographic information; participants were presented with scenario information including the following description:

Suppose that you recently bought milk from a domestic brand on [X] channel (X = online or offline). Now it has been found that the milk you purchased has safety issues and subsequently has been recalled by [Y] initiator (Y = the company, the government, or the news media). Consumers can request to return the product and get a refund.

At the conclusion of presenting this scenario presentation, participants answered the following three questions:

1. If you want to buy milk immediately, which brand of milk would you most likely choose?

2. Are you willing to buy other products from the same brand in the coming month?

3. Will you still buy milk from the recalled channel in the next three months?

The goal is to measure the spillover effects. The first question measures the impact of food recall on the involed product. The second measures the spillover effect of food recall on the recalled brand. The third measures the spillover effect of food recall on the purchasing channel.

### Probit regression

#### Multinomial probit model

Choice of milk brands after the recall crisis, *Milk_brand*, is a categorical dependent variable with outcomes that have no natural ordering. It has three options, and we employ a multinomial probit model to study the impact of recalls on consumer brand choice.

Using the random utility method, it is assumed that the utility generated by participant *i*’s choice of alternative *j* is

1 $${{{\mathrm{U}}}}_{{{{\mathrm{ij}}}}} = {{{\mathrm{x}}}}_i^\prime {\upbeta}_{{{\mathrm{j}}}} + {\upvarepsilon}_{{{{\mathrm{ij}}}}}\left( {{{{\mathrm{i}}}} = 1, \ldots ,{{{\mathrm{I}}}};{{{\mathrm{j}}}} = 1,2, \ldots ,{{{\mathrm{k}}}}} \right)$$

The explanatory variables, x_*i*_, are case-specific or alternative-invariant. The coefficient β_*j*_ indicates that the effect of x_*i*_ on random utility U_*ij*_ depends on alternative *j*. Participant *i* chooses alternative *j* only if the utility of alternative *j* is higher than that of other alternatives, so the probability of participant *i* choosing alternative *j* is

2 $$\begin{array}{*{20}{c}} {\mathrm{P}\left( {{{{\mathrm{Milk}}}}\_{{{\mathrm{brand}}}}_{{{\mathrm{i}}}} = {{{\mathrm{j|x}}}}_{{{\mathrm{i}}}}} \right) = {\mathrm{P}}\left( {{{{\mathrm{U}}}}_{{{{\mathrm{ij}}}}} \ge {{{\mathrm{U}}}}_{{{{\mathrm{ik}}}}},\forall\, {{{\mathrm{k}}}} \,\ne\, {{{\mathrm{j}}}}} \right)} \end{array}$$

where *Milk_brand*_*i*_ represents participant *i*’s choice after the milk recall, and *j* = 0, 1, and 2, with 0 = to not purchase milk; 1 = to purchase milk of another brand; and 2 = to purchase milk of the same brand (set as a base). The explanatory variable x_*i*_ includes the following variables:

3 $$\begin{array}{*{20}{l}} {{{{\mathrm{x}}}}_{{{\mathrm{i}}}} = \left( {\begin{array}{*{20}{l}} {{{{\mathrm{Online}}}},{{{\mathrm{Voluntary}}}},{{{\mathrm{Mandatory}}}},{{{\mathrm{FoodonlinefreqH}}}},{{{\mathrm{MilkfreqH}}}},} \\ {{{{\mathrm{WorryH}}}},{{{\mathrm{Price}}}},{{{\mathrm{Brand}}}},{{{\mathrm{Age}}}},{{{\mathrm{Male}}}},{{{\mathrm{Edu}}}},{{{\mathrm{Income}}}},{{{\mathrm{Have}}}}\_{{{\mathrm{old}}}}} \end{array}} \right),} \end{array}$$

where *Online*, *Voluntary* and *Mandatory* are dummy variables derived based on the settings of the scenario experiments, with *Online* = 1 if milk was purchased on online channel; *Voluntary* = 1 if the milk was recalled by firm voluntarily; *Mandatory* = 1 if milk was recalled by government mandatorily. *FoodonlinefreqH* and *MilkfreqH* are dummy variables representing whether respondents purchase food online and milk frequently, respectively (1 = once a week at least; 0 = otherwise). *WorryH* is a dummy variable measuring whether respondents would be worried about their health if they had consumed food that was recalled. *Price* and *Brand*, using a five-level Likert scale (5 very important, 1 not important at all), measure the importance of price and brand in consumers’ milk purchasing decision. Demographic variables include *Male* (1 = male; 0 = female), *Edu* (years of schooling), *Age*, *Income* (annual household income), and, *Have_old* (whether there are any elderly members in the household: 1 = Yes; 0 = No).

#### Probit model

Whether to buy other products of the same brand, *Other_products*, is a binary dependent variable, and we employ a probit model. Probit models assume that the probability of a positive outcome is determined by the standard normal cumulative distribution function. The two-point distribution probability of *Other_products* for participant *i* is

4 $$\begin{array}{ll} {\left\{ {\begin{array}{ll} {P\left( {{{{\mathrm{Other}}}}\_{{{\mathrm{products}}}}_{{{\mathrm{i}}}} = 1{{{\mathrm{|x}}}}_{{{\mathrm{i}}}}} \right) = F\left( {{{{\mathrm{x}}}}_{{{\mathrm{i}}}},{\upbeta}} \right)} \\ {P\left( {{{{\mathrm{Other}}}}\_{{{\mathrm{products}}}}_{{{\mathrm{i}}}} = 0{{{\mathrm{|x}}}}_{{{\mathrm{i}}}}} \right) = 1 - F\left( {{{{\mathrm{x}}}}_{{{\mathrm{i}}}},{\upbeta}} \right)}, \end{array}} \right.} \end{array}$$

where F(x_i_, β) is the the standard cumulative normal, and then

5 $${\mathrm{P}}\left( {{{{\mathrm{Other}}}}\_{{{\mathrm{products}}}}_{{{\mathrm{i}}}} = 1\left| {{{{\mathrm{x}}}}_{{{\mathrm{i}}}}} \right.} \right) = {\mathrm{F}}\left( {{{{\mathrm{x}}}}_{{{\mathrm{i}}}},{\upbeta}} \right) = \Phi \left( {{{{\mathrm{x}}}}_i^\prime {\upbeta}} \right) = {\int}_{ \!\!- \infty }^{{{{\mathrm{x}}}}_i^\prime {\upbeta}} {\upphi \left( {{{\mathrm{t}}}} \right){{{\mathrm{dt}}}}}$$

*Other_products*_*i*_ = 1 represents participant *i* is willing to purchase other products of the recalled brand. The explanatory variable x_i_ in the probit model is the same as x_i_ in the multinomial probit model.

#### Ordered probit model

Whether to continue buying milk from the purchase channel, *Channel*, is an ordinal variable. For *Channel*, we used a two-level question format. Specifically, we first asked consumers if they would purchase milk again on the channel within one month. If they answered yes, we asked a follow-up question if they would purchase milk again on the channel within one week; if they answered no, the same question is asked with a longer duration, i.e., three months. Thus, we measure the duration that consumers take to restore confidence in milk purchases on the purchasing channel after a recall by using four categories, i.e., one week, one month, three months, and more than three months. Then we employ an ordered probit model to examine consumers’ purchase intention in the original purchasing channel. Suppose the linear function of *Channel* is

6 $${{{\mathrm{Channel}}}}_{{{\mathrm{i}}}}^ \ast = {{{\mathrm{x}}}}_i^\prime \beta + {\upmu}_{{{\mathrm{i}}}}$$

7 $$\begin{array}{ll} {{{{\mathrm{Channel}}}}_{{{\mathrm{i}}}} = \left\{ {\begin{array}{ll} {1,\,if\,three\,months \le {{{\mathrm{Channel}}}}^ \ast } \\ {2,\,if\,one\,month\, < \, {{{\mathrm{Channel}}}}^ \ast \le three\,months} \\ {3,\,if\,one\,week\, < \, {{{\mathrm{Channel}}}}^ \ast \le one\,month} \\ {4,\,if\,{{{\mathrm{Channel}}}}^ \ast \le one\,week} \end{array}} \right.} \end{array}$$

The probability of *Channel* can be estimated as

8 $$\begin{array}{*{20}{c}} {P\left( {{{{\mathrm{Channel}}}}_{{{\mathrm{i}}}} = {{{\mathrm{t}}}}} \right) = P\left( {{{{\mathrm{k}}}}_{{{{\mathrm{t}}}} - 1} \,<\, {\upbeta}_1{{{\mathrm{x}}}}_{1{{{\mathrm{i}}}}} + {\upbeta}_2{{{\mathrm{x}}}}_{2{{{\mathrm{i}}}}} + \ldots + {\upbeta}_{{{\mathrm{k}}}}{{{\mathrm{x}}}}_{{{{\mathrm{ki}}}}} + {\upmu}_{{{\mathrm{i}}}} \le {{{\mathrm{k}}}}_{{{\mathrm{t}}}}} \right)} \end{array}$$

where *μ*_*i*_ is assumed to follow the standard normal distribution. We estimate the coefficients of $\beta _1,\beta _2,...,\beta _k$ together with the cutpoints $k_1,k_2,...,k_{T - 1}$, where *T* is the number of possible outcomes, i.e., 4. Similarly, the explanatory variables x_i_ in the ordered probit model are the same as x_i_ in the multinomial probit model.

## Supplementary information

dataset

code

## Data Availability

The authors declare that the data supporting the findings of this study are available within the paper and supplementary information files. The data also can be available in in the GitHub repository upon request: https://github.com/Yi-Zh1/Spillover-effects-of-food-recalls-A-milk-recall-scenario-experiment-in-China.git.

## Code availability

The authors declare that the code supporting the findings of this study are available within the paper and supplementary information files. The data also can be available in the GitHub repository upon request: https://github.com/Yi-Zh1/Spillover-effects-of-food-recalls-A-milk-recall-scenario-experiment-in-China.git.
