# Supplementary material for: Spillover effects of food recalls: A milk recall scenario experiment in China
Source: NPJ Sci Food. 2022 Apr 22;6:24. doi: 10.1038/s41538-022-00139-1 (PMC9033824; doi:10.1038/s41538-022-00139-1)
Supplement: Supplementary file 2 — code [file 41538_2022_139_MOESM2_ESM.pdf]

/*Regression*/

/*Table 1.Multinomial Probit model results of milk choice*/

```
mprobit Milk_brand i.online o.news i.voluntary i.mandatory o.foodonlinefreqL i.foodonlinefreqH o.milkfreqL i.milkfreqH o.worryL i.worryH price brand age i.male edu income i.have_old , base(2)

mprobit Milk_brand i.online o.news i.voluntary i.mandatory o.online_news i.online_voluntary i.online_mandatory o.foodonlinefreqL i.foodonlinefreqH o.milkfreqL i.milkfreqH o.worryL i.worryH price brand age i.male edu income i.have_old , base(2)
```

/*Table 2.Probit model results of whether purchasing other products of the same brand*/

```
probit Other_products i.online o.news i.voluntary i.mandatory o.foodonlinefreqL i.foodonlinefreqH o.milkfreqL i.milkfreqH o.worryL i.worryH price brand age i.male edu income i.have_old

probit Other_products i.online o.news i.voluntary i.mandatory o.online_news i.online_voluntary i.online_mandatory o.foodonlinefreqL i.foodonlinefreqH o.milkfreqL i.milkfreqH o.worryL i.worryH price brand age i.male edu income i.have_old
```

/*Table 3.Ordered Probit model results of milk purchasing platform*/

```
oprobit Channel i.online o.news i.voluntary i.mandatory o.foodonlinefreqL i.foodonlinefreqH o.milkfreqL i.milkfreqH o.worryL i.worryH price brand age i.male edu income i.have_old

oprobit Channel i.online o.news i.voluntary i.mandatory o.online_news i.online_voluntary i.online_mandatory o.foodonlinefreqL i.foodonlinefreqH o.milkfreqL i.milkfreqH o.worryL i.worryH price brand age i.male edu income i.have_old
```

Note: The analysis software we used is Stata 16.
